# Bis(3-amino­pyrazine-2-carboxyl­ato-κ^2^ *N* ^1^,*O*)diaqua­cobalt(II)

**DOI:** 10.1107/S1600536813002183

**Published:** 2013-01-31

**Authors:** Rafika Bouchene, Sofiane Bouacida, Fadila Berrah, Ratiba Belhouas, Hocine Merazig

**Affiliations:** a Laboratoire de Chimie Appliquée et Technologie des Matériaux LCATM, Université Oum El Bouaghi, Algeria; b Département Sciences de la Matière, Faculté des Sciences Exactes et Sciences de la Nature et de la Vie, Université Oum El Bouaghi, Algeria; c Unité de Recherche de Chimie de l’Environnement et Moléculaire Structurale, CHEMS, Faculté des Sciences Exactes, Université Mentouri Constantine 25000, Algeria

## Abstract

In the title compound, [Co(C_5_H_4_N_3_O_2_)_2_(H_2_O)_2_], the Co^II^ atom is situated on a twofold rotation axis and is *N*,*O*-chelated by two 3-amino­pyrazine-2-carboxyl­ate anions and additionally bonded to the O atoms of two water mol­ecules, leading to a slightly distorted octa­hedral coordination environment. The crystal packing is dominated by inter­molecular O—H⋯O, O—H⋯N and N—H⋯O hydrogen bonding involving the water mol­ecules and amino groups as donors and carboxyl­ate O atoms, as well as the non-coordinating heterocyclic N atoms as acceptors, resulting in a three-dimensional network. An intra­molecular N—H⋯O hydrogen bond is also observed.

## Related literature

For the role of *N,O*-coordination in the crystal structures of metal complexes with pyrazine-2-carboxyl­ate as ligand, see: Alcock *et al.* (1996 ▶); Dong *et al.* (2000 ▶); Kubota *et al.* (2006 ▶); Luo *et al.* (2004 ▶). For related pyrazine-2-carboxyl­ate cobalt(II) complexes and their applications, see: Fan *et al.* (2007 ▶); Liu *et al.* (2007 ▶); McCleverty & Meyer (2004 ▶); Shi *et al.* (2011 ▶); Sun *et al.* (2004 ▶); Tanase *et al.* (2006 ▶). For the influence of hydrogen bonding in related systems, see: Bouacida *et al.* (2007 ▶, 2009 ▶).
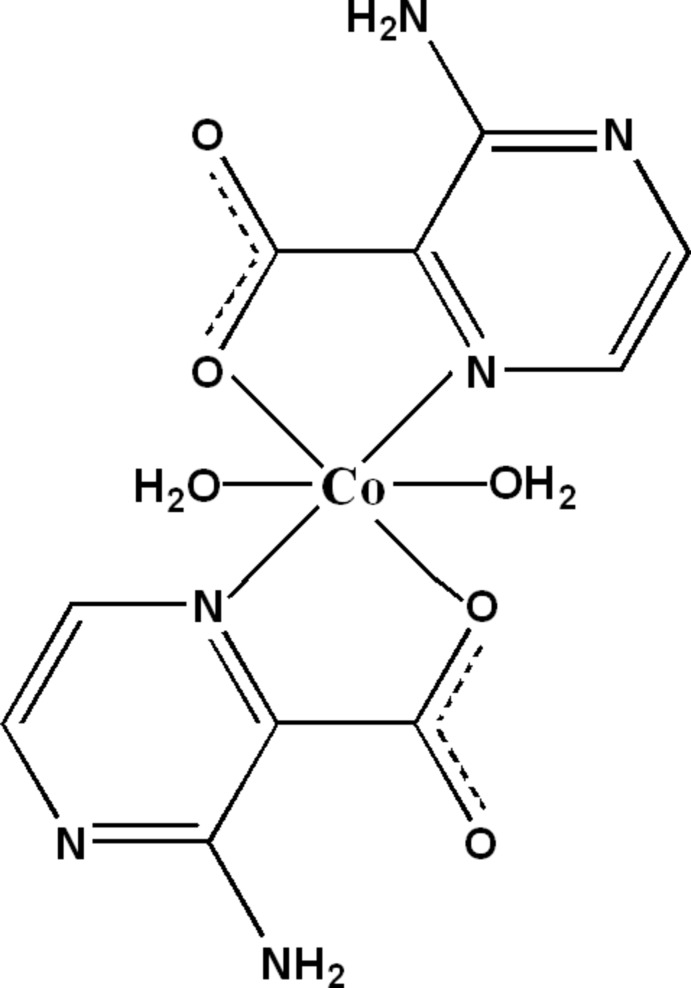


## Experimental

### 

#### Crystal data

[Co(C_5_H_4_N_3_O_2_)_2_(H_2_O)_2_]
*M* *_r_* = 371.19
Monoclinic, 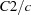

*a* = 7.8823 (2) Å
*b* = 12.7467 (2) Å
*c* = 13.6851 (3) Å
β = 91.918 (1)°
*V* = 1374.22 (5) Å^3^
*Z* = 4
Mo *K*α radiation
μ = 1.29 mm^−1^
*T* = 295 K
0.11 × 0.09 × 0.05 mm

#### Data collection

Bruker APEXII CCD diffractometer
17706 measured reflections
4800 independent reflections
3043 reflections with *I* > 2σ(*I*)
*R*_int_ = 0.041

#### Refinement

*R*[*F* ^2^ > 2σ(*F* ^2^)] = 0.034
*wR*(*F* ^2^) = 0.082
*S* = 0.92
4800 reflections
111 parameters
H atoms treated by a mixture of independent and constrained refinement
Δρ_max_ = 0.48 e Å^−3^
Δρ_min_ = −0.38 e Å^−3^

### 

Data collection: *APEX2* (Bruker, 2011 ▶); cell refinement: *SAINT* (Bruker, 2011 ▶); data reduction: *SAINT*; program(s) used to solve structure: *SIR2002* (Burla *et al.*, 2005 ▶); program(s) used to refine structure: *SHELXL97* (Sheldrick, 2008 ▶); molecular graphics: *ORTEP-3 for Windows* (Farrugia, 2012 ▶) and *DIAMOND* (Brandenburg & Berndt, 2001 ▶); software used to prepare material for publication: *WinGX* (Farrugia, 2012 ▶).

## Supplementary Material

Crystal structure: contains datablock(s) global, I. DOI: 10.1107/S1600536813002183/wm2721sup1.cif

Structure factors: contains datablock(s) I. DOI: 10.1107/S1600536813002183/wm2721Isup2.hkl

Additional supplementary materials:  crystallographic information; 3D view; checkCIF report

## Figures and Tables

**Table 1:** Hydrogen-bond geometry (Å, °)

*D*—H⋯*A*	*D*—H	H⋯*A*	*D*⋯*A*	*D*—H⋯*A*
O1*W*—H1*W*⋯N2^i^	0.761 (18)	2.070 (18)	2.8254 (12)	172.2 (17)
O1*W*—H2*W*⋯O52^ii^	0.762 (18)	1.898 (17)	2.6470 (12)	167.1 (16)
N3—H3*A*⋯O51^iii^	0.86	2.33	3.0525 (12)	141
N3—H3*B*⋯O52	0.86	2.07	2.7036 (13)	130

Symmetry codes: (i) 
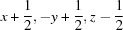
; (ii) 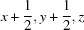; (iii) 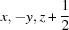.

**Fig. 1.**
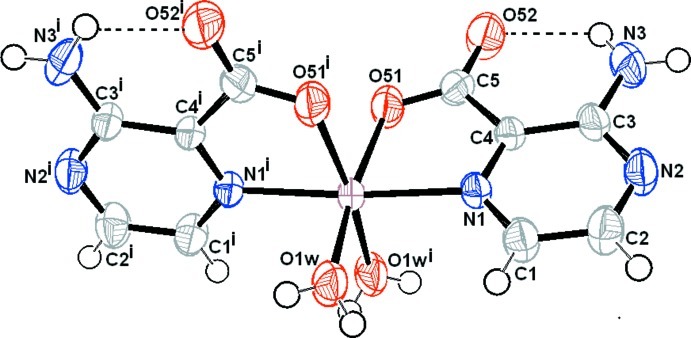
A view of the coordination environment of the CoII atom of (I) with the atom-labelling scheme. Displacement ellipsoids are drawn at the 50% probability level. H atoms are represented as small spheres of arbitrary radius. [Symmetry code: (i)-x, y, -z + 3/2.]

**Fig. 2.**
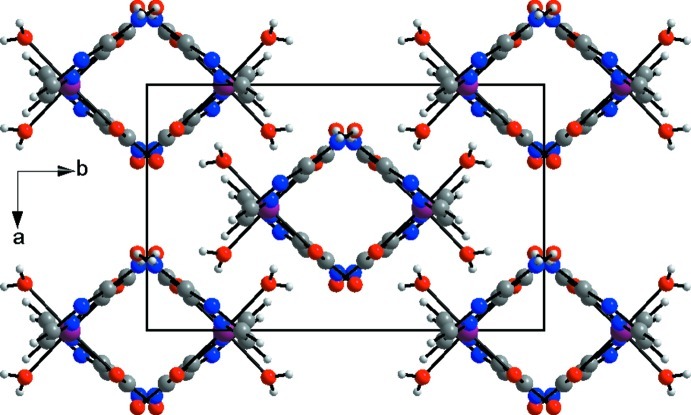
The packing of the structure of (I) viewed along the c axis

**Fig. 3.**
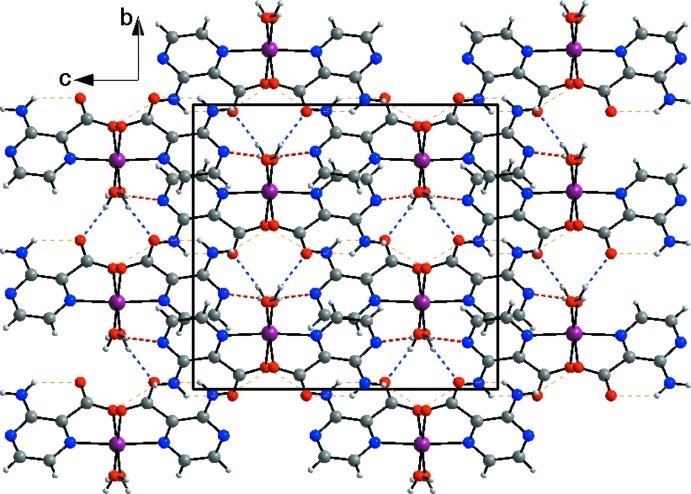
Hydrogen bonding interactions (dashed lines) in the structure of (I)

## supplementary crystallographic information

### Comment

During our recent research in the field of *N,O*-donor stabilized metal complexes we have prepared the title compound. As ligand we have chosen pyrazine-2-carboxylate that already has been extensively studied (Alcock *et al.*, 1996; Dong *et al.*, 2000; Kubota *et al.*, 2006; Luo *et al.*, 2004; Shi *et al.*, 2011; Fan *et al.*, 2007; Liu *et al.*, 2007). Some of its cobalt(II) complexes have also been reported for multitude applications (Tanase *et al.*, 2006; Sun *et al.*, 2004; McCleverty & Meyer, 2004).

In continuation of our investigations on the influence of hydrogen bonds on the structural features (Bouacida *et al.*, 2007,2009), we report here the crystal growth and crystal structure of the title compound, [Co(C_5_H_4_N_3_O_2_)_2_(H_2_O)_2_] (I).

The asymmetric unit of (I) consists of one-half of the complex molecule, with the other half being generated by a twofold rotation axis running through the Co^II^ atom (Wyckoff site 4 *e*). The latter is octahedrally coordinated by two 3-aminopyrazine-2-carboxylate anions acting in a bidentate manner and by two water molecules. The molecular geometry and the atom-numbering scheme of (I) are shown in Fig. 1.

Bond lengths and angles observed in the different entities show normal features and are consistent with those reported previously for related systems (Shi *et al.*, 2011). Fig. 2 shows a packing diagram of the structure. Parallel to the *c* axis channels with a square cross-section are formed. The crystal packing can be described by stacking of alternating layers parallel to (110). The layers are linked together by O1W—H···N, O1W—H···O and N—H···O interactions involving the water molecules and amino functions as donors and carboxylate O atoms as well as the non-coordinating heterocyclic N atoms as acceptors (Fig. 3, Table 1). These interactions lead to the formation of a three-dimensional network.

### Experimental

The title compound was obtained from a mixture of cobalt(II) chloride hexahydrate (0.05 g, 0.2 mmol), 3-aminopyrazine-2-carboxylic acid (0.03 g, 0.2 mmol) and acidified water (25 ml, HCl 37%). The solution was evaporated at room temperature for two weeks. Yellow single crystals were obtained and were carefully isolated under a polarizing microscope for analysis by X-ray diffraction.

### Refinement

The H atoms were localized in Fourier maps but were eventually introduced in calculated positions and treated as riding on their parent atoms (C or N) with C—H = 0.93 Å and N—H = 0.86 Å with *U*_iso_(H) = 1.2*U*_eq_(C or N). The water H atoms H1W and H2W were also located in a difference Fourier map. Their positions were refined freely, but their temperature factors were refined isotropically with *U*_iso_(H) = 1.5*U*_eq_(OW).

### Crystal data

**Table:**

[Co(C_5_H_4_N_3_O_2_)_2_(H_2_O)_2_]	*F*(000) = 756
*M**_r_* = 371.19	*D*_x_ = 1.794 Mg m^−^^3^
Monoclinic, *C*2/*c*	Mo *K*α radiation, λ = 0.71073 Å
*a* = 7.8823 (2) Å	Cell parameters from 6448 reflections
*b* = 12.7467 (2) Å	θ = 3.0–38.7°
*c* = 13.6851 (3) Å	µ = 1.29 mm^−^^1^
β = 91.918 (1)°	*T* = 295 K
*V* = 1374.22 (5) Å^3^	Block, yellow
*Z* = 4	0.11 × 0.09 × 0.05 mm

### Data collection

**Table:**

Bruker APEXII CCD diffractometer	3043 reflections with *I* > 2σ(*I*)
Radiation source: sealed tube	*R*_int_ = 0.041
Graphite monochromator	θ_max_ = 42.1°, θ_min_ = 3.0°
φ and ω scans	*h* = −14→10
17706 measured reflections	*k* = −24→22
4800 independent reflections	*l* = −25→16

### Refinement

**Table:**

Refinement on *F*^2^	Primary atom site location: structure-invariant direct methods
Least-squares matrix: full	Secondary atom site location: difference Fourier map
*R*[*F*^2^ > 2σ(*F*^2^)] = 0.034	Hydrogen site location: inferred from neighbouring sites
*wR*(*F*^2^) = 0.082	H atoms treated by a mixture of independent and constrained refinement
*S* = 0.92	*w* = 1/[σ^2^(*F*_o_^2^) + (0.0413*P*)^2^] where *P* = (*F*_o_^2^ + 2*F*_c_^2^)/3
4800 reflections	(Δ/σ)_max_ = 0.001
111 parameters	Δρ_max_ = 0.48 e Å^−^^3^
0 restraints	Δρ_min_ = −0.38 e Å^−^^3^

### Special details

**Table:**

Geometry. All e.s.d.'s (except the e.s.d. in the dihedral angle between two l.s. planes) are estimated using the full covariance matrix. The cell e.s.d.'s are taken into account individually in the estimation of e.s.d.'s in distances, angles and torsion angles; correlations between e.s.d.'s in cell parameters are only used when they are defined by crystal symmetry. An approximate (isotropic) treatment of cell e.s.d.'s is used for estimating e.s.d.'s involving l.s. planes.
Refinement. Refinement of *F*^2^ against ALL reflections. The weighted *R*-factor *wR* and goodness of fit *S* are based on *F*^2^, conventional *R*-factors *R* are based on *F*, with *F* set to zero for negative *F*^2^. The threshold expression of *F*^2^ > σ(*F*^2^) is used only for calculating *R*-factors(gt) *etc*. and is not relevant to the choice of reflections for refinement. *R*-factors based on *F*^2^ are statistically about twice as large as those based on *F*, and *R*- factors based on ALL data will be even larger.

### Fractional atomic coordinates and isotropic or equivalent isotropic displacement parameters (Å^2^)

**Table:**

	*x*	*y*	*z*	*U*_iso_*/*U*_eq_	
Co1	0	0.195326 (13)	0.75	0.02207 (5)	
O1W	0.19025 (11)	0.30652 (6)	0.76033 (6)	0.03609 (17)	
H1W	0.250 (2)	0.3194 (11)	0.7195 (13)	0.054*	
H2W	0.1779 (19)	0.3585 (14)	0.7867 (12)	0.054*	
O51	−0.18087 (10)	0.07591 (6)	0.76347 (5)	0.03603 (17)	
O52	−0.31276 (13)	−0.02336 (7)	0.87118 (6)	0.0538 (2)	
N1	−0.03120 (10)	0.18853 (6)	0.90389 (5)	0.02230 (13)	
N2	−0.09937 (11)	0.16410 (8)	1.09943 (6)	0.03211 (17)	
C1	0.04090 (12)	0.25148 (8)	0.97172 (6)	0.02822 (18)	
H1	0.1164	0.3035	0.9535	0.034*	
N3	−0.26554 (13)	0.02032 (8)	1.06349 (7)	0.0453 (2)	
H3A	−0.2827	0.0138	1.1249	0.054*	
H3B	−0.3112	−0.0232	1.0224	0.054*	
C2	0.00267 (13)	0.23880 (9)	1.06893 (7)	0.0327 (2)	
H2	0.0508	0.2847	1.1148	0.039*	
C3	−0.16811 (12)	0.09790 (7)	1.03189 (7)	0.02792 (18)	
C4	−0.13532 (11)	0.11308 (7)	0.93095 (6)	0.02309 (15)	
C5	−0.21616 (13)	0.04957 (8)	0.84893 (7)	0.03098 (19)	

### Atomic displacement parameters (Å^2^)

**Table:**

	*U*^11^	*U*^22^	*U*^33^	*U*^12^	*U*^13^	*U*^23^
Co1	0.02920 (9)	0.02103 (9)	0.01636 (7)	0	0.00654 (5)	0
O1W	0.0437 (4)	0.0371 (4)	0.0284 (3)	−0.0159 (3)	0.0152 (3)	−0.0072 (3)
O51	0.0514 (4)	0.0342 (4)	0.0230 (3)	−0.0146 (3)	0.0090 (3)	−0.0052 (3)
O52	0.0808 (6)	0.0417 (5)	0.0405 (4)	−0.0356 (4)	0.0263 (4)	−0.0128 (4)
N1	0.0249 (3)	0.0230 (3)	0.0193 (3)	0.0009 (3)	0.0057 (2)	0.0015 (2)
N2	0.0378 (4)	0.0383 (4)	0.0207 (3)	0.0010 (4)	0.0071 (3)	0.0019 (3)
C1	0.0285 (4)	0.0338 (5)	0.0225 (4)	−0.0058 (4)	0.0025 (3)	0.0005 (3)
N3	0.0686 (7)	0.0378 (5)	0.0307 (4)	−0.0167 (5)	0.0196 (4)	0.0032 (4)
C3	0.0343 (4)	0.0259 (4)	0.0242 (4)	0.0031 (3)	0.0112 (3)	0.0041 (3)
C5	0.0410 (5)	0.0242 (4)	0.0284 (4)	−0.0072 (4)	0.0117 (4)	−0.0050 (3)
C4	0.0285 (4)	0.0202 (4)	0.0210 (3)	0.0010 (3)	0.0083 (3)	0.0014 (3)
C2	0.0348 (5)	0.0411 (6)	0.0221 (4)	−0.0031 (4)	0.0007 (3)	−0.0024 (4)

### Geometric parameters (Å, º)

**Table:**

Co1—O1W	2.0648 (8)	N1—C1	1.3390 (11)
Co1—O1W^i^	2.0648 (8)	N2—C2	1.3230 (14)
Co1—O51	2.0979 (7)	N2—C3	1.3515 (13)
Co1—O51^i^	2.0979 (7)	C1—C2	1.3834 (13)
Co1—N1^i^	2.1303 (7)	C1—H1	0.93
Co1—N1	2.1303 (7)	N3—C3	1.3329 (13)
O1W—H1W	0.760 (18)	N3—H3A	0.86
O1W—H2W	0.763 (18)	N3—H3B	0.86
O51—C5	1.2568 (12)	C3—C4	1.4270 (12)
O52—C5	1.2460 (12)	C5—C4	1.5078 (13)
N1—C4	1.3252 (11)	C2—H2	0.93
			
O1W—Co1—O1W^i^	93.31 (5)	C1—N1—Co1	126.91 (6)
O1W—Co1—O51	170.46 (3)	C2—N2—C3	117.88 (8)
O1W^i^—Co1—O51	90.57 (4)	N1—C1—C2	119.71 (9)
O1W—Co1—O51^i^	90.57 (4)	N1—C1—H1	120.1
O1W^i^—Co1—O51^i^	170.46 (3)	C2—C1—H1	120.1
O51—Co1—O51^i^	86.97 (5)	C3—N3—H3A	120
O1W—Co1—N1^i^	89.31 (3)	C3—N3—H3B	120
O1W^i^—Co1—N1^i^	93.89 (3)	H3A—N3—H3B	120
O51—Co1—N1^i^	99.13 (3)	N3—C3—N2	117.61 (8)
O51^i^—Co1—N1^i^	77.43 (3)	N3—C3—C4	122.66 (9)
O1W—Co1—N1	93.89 (3)	N2—C3—C4	119.73 (8)
O1W^i^—Co1—N1	89.31 (3)	O52—C5—O51	125.65 (9)
O51—Co1—N1	77.43 (3)	O52—C5—C4	117.72 (8)
O51^i^—Co1—N1	99.13 (3)	O51—C5—C4	116.63 (8)
N1^i^—Co1—N1	175.34 (4)	N1—C4—C3	120.19 (8)
Co1—O1W—H1W	124.2 (12)	N1—C4—C5	115.59 (7)
Co1—O1W—H2W	121.7 (11)	C3—C4—C5	124.20 (8)
H1W—O1W—H2W	104.7 (15)	N2—C2—C1	122.81 (9)
C5—O51—Co1	116.60 (6)	N2—C2—H2	118.6
C4—N1—C1	119.58 (7)	C1—C2—H2	118.6
C4—N1—Co1	113.50 (6)		
			
O1W^i^—Co1—O51—C5	−93.84 (8)	Co1—O51—C5—O52	−175.74 (10)
O51^i^—Co1—O51—C5	95.39 (8)	Co1—O51—C5—C4	4.97 (12)
N1^i^—Co1—O51—C5	172.13 (8)	C1—N1—C4—C3	−1.05 (13)
N1—Co1—O51—C5	−4.67 (7)	Co1—N1—C4—C3	179.46 (6)
O1W—Co1—N1—C4	−172.54 (6)	C1—N1—C4—C5	177.36 (8)
O1W^i^—Co1—N1—C4	94.19 (6)	Co1—N1—C4—C5	−2.13 (10)
O51—Co1—N1—C4	3.45 (6)	N3—C3—C4—N1	−176.88 (9)
O51^i^—Co1—N1—C4	−81.34 (6)	N2—C3—C4—N1	3.34 (13)
O1W—Co1—N1—C1	8.01 (8)	N3—C3—C4—C5	4.85 (15)
O1W^i^—Co1—N1—C1	−85.25 (8)	N2—C3—C4—C5	−174.93 (9)
O51—Co1—N1—C1	−175.99 (8)	O52—C5—C4—N1	178.82 (10)
O51^i^—Co1—N1—C1	99.22 (8)	O51—C5—C4—N1	−1.84 (13)
C4—N1—C1—C2	−1.63 (14)	O52—C5—C4—C3	−2.84 (15)
Co1—N1—C1—C2	177.78 (7)	O51—C5—C4—C3	176.50 (9)
C2—N2—C3—N3	177.46 (9)	C3—N2—C2—C1	0.07 (16)
C2—N2—C3—C4	−2.76 (14)	N1—C1—C2—N2	2.23 (16)

Symmetry code: (i) −*x*, *y*, −*z*+3/2.

### Hydrogen-bond geometry (Å, º)

**Table:**

*D*—H···*A*	*D*—H	H···*A*	*D*···*A*	*D*—H···*A*
O1*W*—H1*W*···N2^ii^	0.761 (18)	2.070 (18)	2.8254 (12)	172.2 (17)
O1*W*—H2*W*···O52^iii^	0.762 (18)	1.898 (17)	2.6470 (12)	167.1 (16)
N3—H3*A*···O51^iv^	0.86	2.33	3.0525 (12)	141
N3—H3*B*···O52	0.86	2.07	2.7036 (13)	130
C1—H1···O52^iii^	0.93	2.55	3.4010 (13)	153

Symmetry codes: (ii) *x*+1/2, −*y*+1/2, *z*−1/2; (iii) *x*+1/2, *y*+1/2, *z*; (iv) *x*, −*y*, *z*+1/2.

## References

[bb1] Alcock, N. W., Kemp, T. J., Marc Roe, S. & Leciejewicz, J. (1996). *Inorg. Chim. Acta*, **248**, 241–246.

[bb2] Bouacida, S., Belhouas, R., Kechout, H., Merazig, H. & Bénard-Rocherullé, P. (2009). *Acta Cryst.* E**65**, o628–o629. 10.1107/S1600536809006072 PMC2968595 21582279

[bb3] Bouacida, S., Merazig, H., Benard-Rocherulle, P. & Rizzoli, C. (2007). *Acta Cryst.* E**63**, m379–m381.

[bb4] Brandenburg, K. & Berndt, M. (2001). *DIAMOND* Crystal Impact, Bonn, Germany.

[bb5] Bruker (2011). *APEX2* and *SAINT* Bruker AXS Inc., Madison, Wisconsin, USA.

[bb6] Burla, M. C., Caliandro, R., Camalli, M., Carrozzini, B., Cascarano, G. L., De Caro, L., Giacovazzo, C., Polidori, G. & Spagna, R. (2005). *J. Appl. Cryst.* **38**, 381–388.

[bb7] Dong, Y.-B., Smith, M. D. & zur Loye, H.-C. (2000). *Inorg. Chem.* **39**, 1943–1949. 10.1021/ic991475y 11428114

[bb8] Fan, G., Chen, S.-P. & Gao, S.-L. (2007). *Acta Cryst.* E**63**, m772–m773.

[bb9] Farrugia, L. J. (2012). *J. Appl. Cryst.* **45**, 849–854.

[bb10] Kubota, Y., Takata, M., Matsuda, R., Kitaura, R., Kitagawa, S. & Kobayashi, T. C. (2006). *Angew. Chem. Int. Ed.* **45**, 4932–4936. 10.1002/anie.200600976 16807951

[bb11] Liu, F.-Y., Shang, R.-L., Du, L., Zhao, Q.-H. & Fang, R.-B. (2007). *Acta Cryst.* E**63**, m120–m122.

[bb12] Luo, J., Alexander, B., Wagner, T. R. & Maggard, P. A. (2004). *Inorg. Chem.* **43**, 5537–5542. 10.1021/ic049609h 15332804

[bb13] McCleverty, J. A. & Meyer, T. J. (2004). *Comprehensive Coordination Chemistry II. From Biology to Nanotechnology*, Vol. 6, *Transition Metal Groups 9–12*, pp. 99–120. Amsterdam: Elsevier Pergamon.

[bb14] Sheldrick, G. M. (2008). *Acta Cryst.* A**64**, 112–122. 10.1107/S0108767307043930 18156677

[bb15] Shi, Q.-Y., Zhang, G.-C., Zhou, C.-S. & Yang, Q. (2011). *Acta Cryst.* E**67**, m1430. 10.1107/S1600536811038591 PMC3201376 22058711

[bb16] Sun, W.-H., Tang, X., Gao, T., Wu, B., Zhang, W. & Ma, H. (2004). *Organometallics*, **23**, 5037–5041.

[bb17] Tanase, S., Martin, V. S., Van Albada, G. A., DeGelder, R., Bouwman, E. & Reedijk, J. (2006). *Polyhedron*, **25**, 2967–2975.

